# Improving injection safety practices of Cambodian healthcare workers through training

**DOI:** 10.1371/journal.pone.0241176

**Published:** 2020-10-30

**Authors:** Udhayashankar Kanagasabai, Adarshpal Singh, Ray W. Shiraishi, Vanthy Ly, Chhaily Hy, Sou Sanith, Sok Srun, Sim Sansam, S. Teak SopHeap, Yuliang Liu, Gerald Jones, Ugonna C. Ijeoma, Naomi Bock, Irene Benech, Dejana Selenic, Bakary Drammah, Renuka Gadde, Fatima D. Mili

**Affiliations:** 1 Epidemic Intelligence Service, CDC, DGHT, Atlanta, GA, United States of America; 2 Becton, Dickinson and Company, Franklifn Lakes, NJ, United States of America; 3 Division of Global HIV/AIDS and Tuberculosis, CDC, Atlanta, GA, United States of America; 4 U.S. Centers for Disease Control and Prevention, Cambodia; 5 Ministry of Health, Phnom Pehn, Cambodia; 6 Pursat Provincial Health Department, Pursat, Cambodia; 7 Division of Health Informatics and Surveillance, Epidemiology and Laboratory Services, CDC, Atlanta, GA, United States of America; National University of Singapore, SINGAPORE

## Abstract

**Background:**

This study evaluated the impact of a safe injection safety training on healthcare worker (HCW) practice and knowledge following an HIV outbreak in Roka commune, Cambodia.

**Methods:**

Surveys were conducted at baseline (September 2016) and seven months after a training intervention (March 2018) using the World Health Organization standardized injection practices assessment tool. HCWs were sampled at 15 purposively government health facilities in two provinces. HCWs were observed during injection practices and interviewed by trained experts from Becton-Dickinson and the Ministry of Health Cambodia. The Rao-Scott chi square test was used test for differences between baseline and follow-up.

**Results:**

We completed 115 observations of practice at baseline and 206 at post-training follow-up. The proportion of patients whose identification was confirmed by HCWs prior to procedure being performed increased from 40.4% to 98% (p <0.0001). The proportion of HCWs who practiced correct hand hygiene increased from 22.0% to 80.6% (p = 0.056) [therapeutic observations] and 17.2% to 63.4% (p = 0.0012) [diagnostic observations]. Immediate disposal of sharps by HCWs decreased from 96.5% to 92.5% (p = 0.0030).

**Conclusions:**

We found significant improvements in the practice of patient identity confirmation and hand hygiene but not in the immediate disposal of sharps in the post-training intervention. However, findings are not representative of all HCWs in the country. Further pre-service and in-service training and monitoring are necessary to ensure sustained behavior change.

## Introduction

Injection overuse and unsafe injection practices have been reported across the world particularly in transitional and low-resource settings [1]. The World Health Organization (WHO) estimates that at least 8–12 billion syringes are sold each year, with an average of 3.7 injections received per person in low-income countries per year [2,3]. Unsafe injection practices such as the re-use of unsterile needles and syringes contribute substantially to the global burden of blood-borne pathogens [4,5]. In 2000, it was estimated that 32% of hepatitis B (HBV), 40% of hepatitis C (HCV), and 5% of HIV infections were caused by contaminated injections [6]. These chronic infections lead to a high burden of morbidity, mortality and cost the world US$ 535 million per year in direct medical expenditures [7]. Unsafe injection practices not only harm the patient but also carry a significant risk to the healthcare worker (HCW) [8]. Healthcare providers are often at risk of encountering needle stick injuries (NSI) while providing patient care [9].

WHO defines a safe injection as one that does not harm the recipient, does not expose the provider to any avoidable risk, and does not result in waste that is dangerous to other people [3]. Unsafe injections practices include, but are not limited to, reuse of syringes for multiple patients or to access shared medications, administration of medication from a single-dose/single-use vial to multiple patients, and failure to use aseptic technique when preparing and administering injections [10].

Safe injection practices involve the administration of rational injection use by a qualified and well-trained person using a sterile device (syringe, needle, etc. that is taken from a sealed, unopened package), adopting sterile technique, and discarding the used devices in a puncture-proof, specially-designed container for safe disposal. HCWs in low-income countries inconsistently practice universal precautions and are commonly exposed to blood in their work via NSI, splash incidents, and direct contact [11]. About thirty different infectious diseases can be transmitted by NSI among which the chances of acquiring HBV infection is far higher than the others [8]. WHO estimates that 66,000 HBV, 16,000 HCV, and 200–5,000 HIV infections each year are caused by occupational exposure with 90% occurring in the low resource settings [11].

### Setting

Cambodia is located in Southeast Asia and has a growing population of both youth and the elderly, with a third currently under 15 years of age. Public health facilities in Cambodia include: (a) health centers, which provide basic health services through a minimum package of activities; (b) provincial and district Referral Hospitals, which provide a complimentary package of activities (CPA) at three levels [CPA-1, CPA-2, CPA-3] based on the composition of staff, number of beds, standard drug kit, and clinical activities; and (c) National Hospitals which provide higher-level tertiary care [12]. In rural areas of Cambodia, only 15% of primary care consultations occur in the public sector. Private non-medical (designated as unqualified by the Cambodian Ministry of Health (MoH)) providers account for half of all healthcare providers [12]. Cambodian’s receive 0.8–5.9 therapeutic injections per person per year, one of the highest reported rates worldwide [13].

Between 2014 and 2015, an outbreak of 242 new HIV cases was reported in Roka Commune, Battambang Province, Cambodia [13,14]. The MoH of Cambodia and the National Center for HIV/AIDS, Dermatology and Sexually Transmitted Diseases (NCHADS) conducted a case-control study determining that cases were 5 times more likely to have received therapeutic injections and ruled out associations with commercial sex work, injection drug use, or blood transfusion [15]. Recommendations from the rapid assessment included education of healthcare workers and communities at large on safe injection practices.

To identify potential gaps in safe injection practices, the Cambodia MoH partnered with the United States Centers for Disease Control and Prevention (CDC) and the medical technology company Becton Dickinson and Company (BD) to conduct a rapid assessment of injection practices at public health facilities [13]. Findings from the rapid assessment were used to implement an HCW training on injection safety best practices in July-September 2017, and a follow-up post assessment in March 2018 to measure changes in practice.

## Methods

We conducted a pre/post evaluation of a training on injection safety best practices. Baseline data were collected from 15 purposively selected public health facilities in Battambang and Pursat provinces in September 2016 [16]. Follow-up data were collected at the same 15 public health facilities in March 2018, approximately 7–8 months after the training. A WHO standardized injection practices assessment tool designed to observe and interview HCWs was used to observe all injections administered and interview licensed HCWs including physicians, nurses, and laboratory technicians [17]. Injection technique was assessed using a standardized checklist [17]. The interview questions ascertained knowledge, attitudes, and practices regarding injection use and safety. The interviews were conducted by injection safety experts accompanied by local translators who translated in real time.

The training curriculum on injections, infusions, and phlebotomy was developed by BD with technical assistance from CDC and context-specific input from Cambodia (MoH) through a Public Private Partnership. All training material were translated into Khmer and translated back to ensure accuracy of translations. The trainings were all conducted by injection safety experts with the assistance of professional translators. In July 2017, a one-week combined didactic and skills based training on injections and infusions was completed, and in September 2017, a three-day training using both didactic and skills based methods on phlebotomy was completed, with subsequent cascade (master trainers who trained fellow HCWs) training of HCWs across the country. A cascade model of professional development which is known to bring about large-scale change was adopted for this training intervention [18]. The cascade model uses a set of master trainers who then subsequently train others, eventually leading to the entire target population receiving the training.

The objectives of the study were to describe the practices of administering injections; assess the knowledge, attitudes, and practice of HCWs regarding injections; and to measure the changes in practice following the training intervention.

### Sampling

We purposively sampled 15 public health facilities from three operational districts (Battambang, Sampov Meas, and Sangke) in Battambang and Pursat provinces. The health facilities were selected in collaboration with the Cambodian MoH using the following criteria:

- Their proximity to the site of the 2014 HIV outbreak
- Provinces that had reported higher injection rates per “Demographic Health Survey 2014” [16]
- Health facilities where injections and phlebotomy procedures are performed daily
- Health facilities where the cascade training intervention was implemented

The target population was HCWs involved in providing injections, collecting and managing medical waste and their supervisors at the selected health facilities in the three operational districts. At referral hospitals, a minimum of eight injections per ward and at health centers a minimum of four injections were observed. All HCWs performing injections or phlebotomy procedures and one supervisor per ward during the survey period were interviewed. Sample size calculations were based on the number of events rather than the number of providers. The total number of events possible per all wards, per facility was used to determine the number of events observed and the number of providers interviewed. The research team did not collect any personally identifying information at baseline or follow-up so some HCWs were likely assessed at both baseline and follow-up.

### Data collection

Data were collected using a standardized tool from WHO and CDC adapted to the context of the Cambodian health system for rapid assessment of injection practices. The baseline survey was conducted using paper assessment forms, while the follow-up survey data were collected using Epi Info™ version 7.2.2.6 software installed on tablet computers. The questionnaires were created in English and the interviews were conducted by trained experts from BD and professional translators who translated them into Khmer in real-time.

### Ethics

The National Ethics Committee for Health Research of the Kingdom of Cambodia approved the protocol. The protocol was reviewed in accordance with the Centers for Disease Control and Prevention (CDC) human research protection procedures and was determined to be research, but CDC investigators did not interact with human subjects nor have access to identifiable data or specimens for research purposes. Informed written consent was obtained from all study participants prior to interviews and no personal identifying information was collected.

### Data analysis

The responses to the questionnaires were entered into an Epi Info™ (version 7.2.2.6 database, CDC 2017). Statistical analysis was performed using Stata/SE 15 (StataCorp. 2017. *Stata Statistical Software*: *Release 15*. College Station, TX: StataCorp LLC). Rao-Scott chi-square test was used to test for differences between baseline and post-training follow-up. Analyses accounted for facility-level clustering.

## Results

Fifteen health facilities were surveyed at baseline (2016) and again at follow-up (2018). These facilities included one referral hospital and three health facilities in Sampov Meas district; one referral hospital and six health centers in Battambang district; and four health centers in Sangke district.

In 2016, 115 injection events were observed compared with 182 in 2018. In 2016, a total of 39 injection providers, 26 injection provider supervisors, and 15 waste handlers were interviewed compared with 71, 24, and 11 in 2018, respectively (Table 1). There could be a recall bias when HCWs and patients are answering questions on blood drawing or injections incidents/experiences of the previous six months. We controlled for this by including options such as “other”, “don’t know” or “don’t recall” and also keeping open ended questions to a minimum. There could also be observer effect as HCWs may improve their performance or behavior if they are aware, they are being observed. We attempted to control for this by ensuring the data collectors do not display any verbal or nonverbal cues (either positive or negative) while observing the procedures or conducting the interviews.

**Table 1:** Summary of pre- and post-training follow-up observations and interviews.

Components	Pre (n)	Post (n)
Facilities	15	15
**Observation**		
Procedures observed*	115	182
Diagnostic	29	41
Therapeutic	86	141
Facility observation	15	15
**Interviews**		
Interview with injection providers	39	71
Interview with supervisors	26	24
Interview with waste handlers	15	11

*Observations did not include immunizations.

### Procedures affecting patient safety

**Fig 1:**
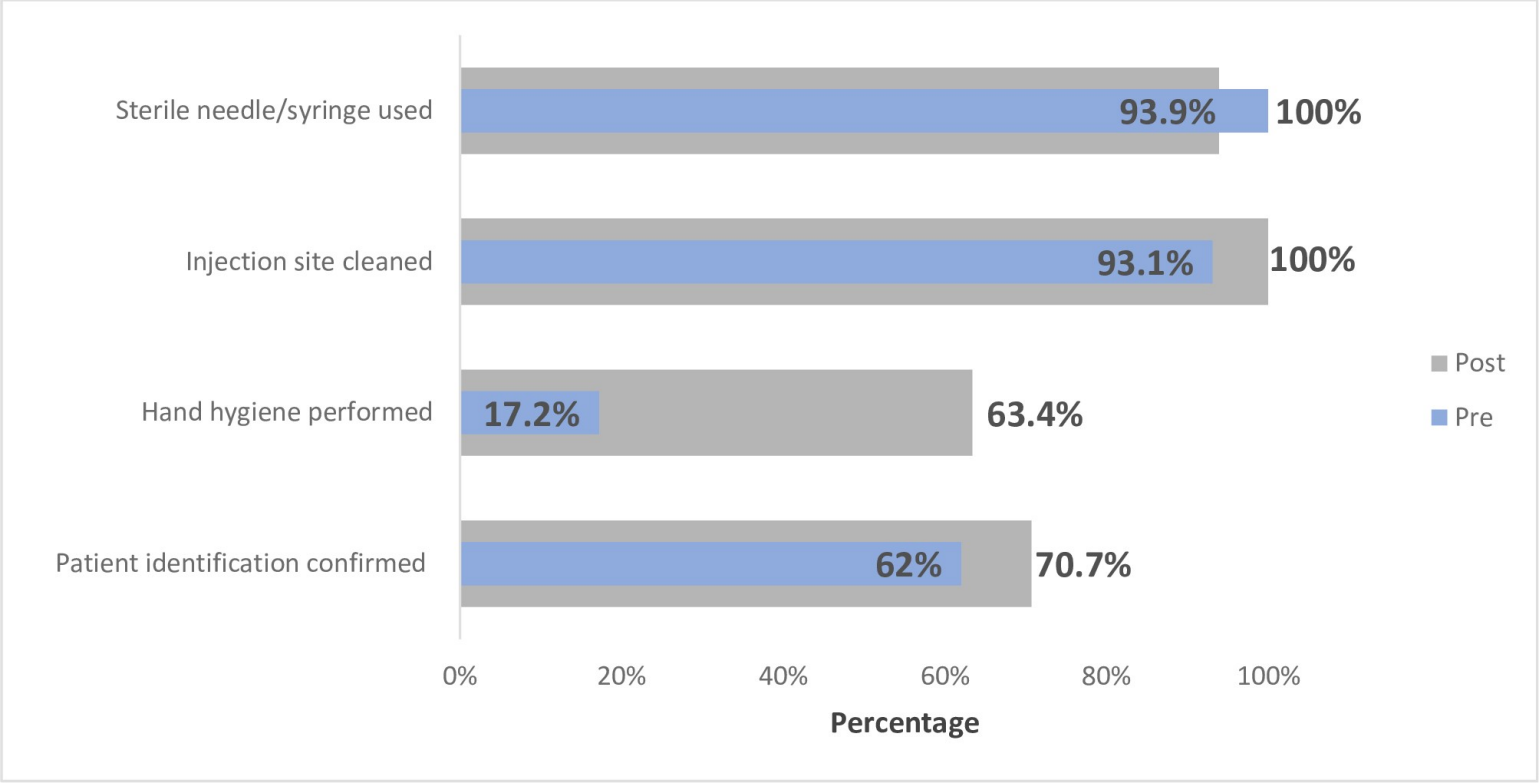
Combined pre and post training assessment of safe injection practices affecting patients (Diagnostic observation).

**Fig 2:**
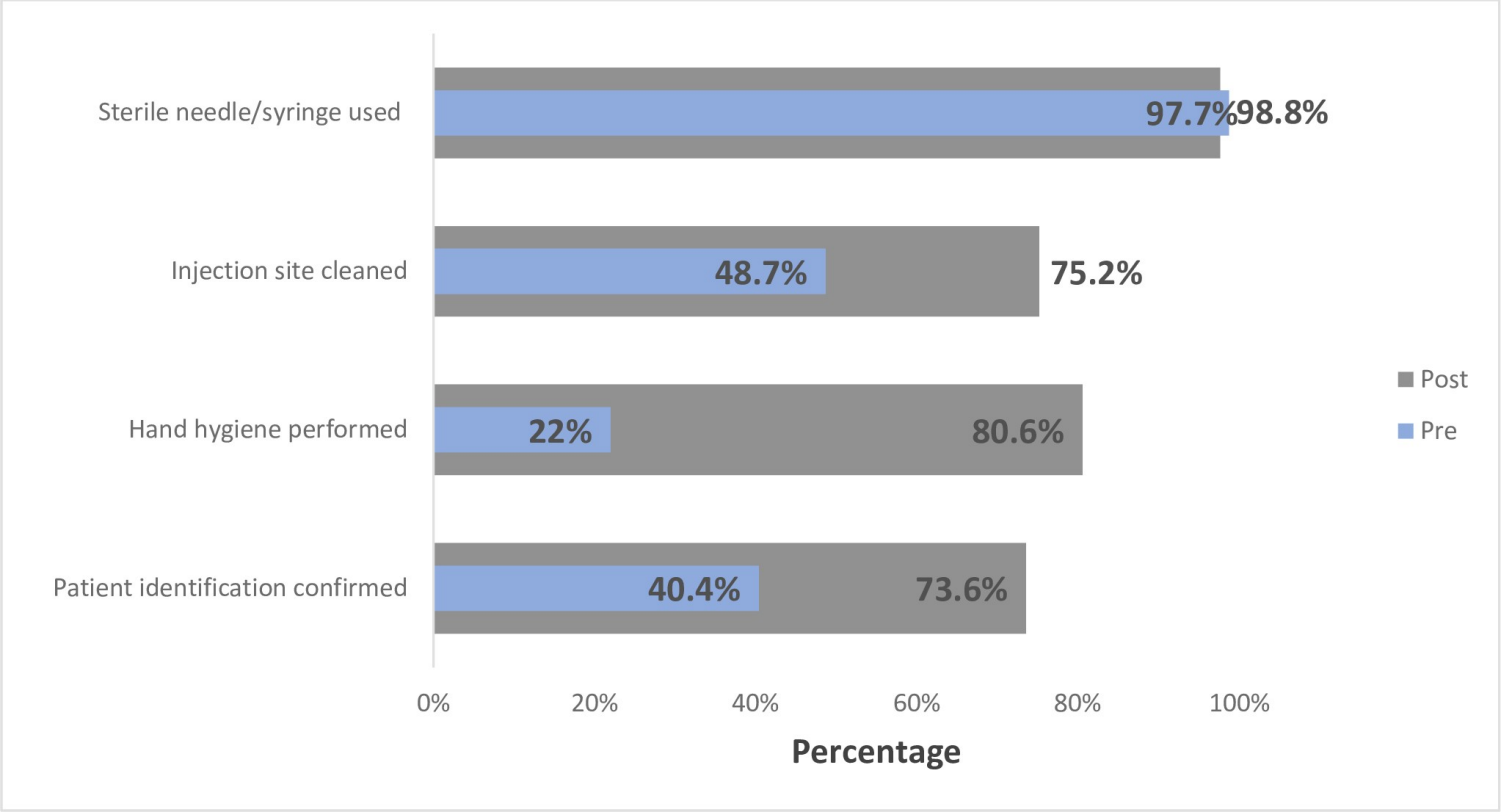
Combined pre- and post-training assessment of safe injection practices affecting patients (Therapeutic observations).

Hand hygiene performed prior to procedures showed significant improvement from baseline to the post-training follow-up (17.2% vs. 63.4%; *p* = 0.0012; Table 2; Fig 1) among diagnostic observations and (22.0% vs. 80.6%; *p =* 0.0056; Table 2; Fig 1). The percentage of HCWs who cleaned an injection site prior to a diagnostic procedure was higher at the post-training follow-up (93.1% baseline vs. 100% post training); however, this increase was not statistically significant (*p* = 0.2786; Fig 1). Among therapeutic procedures, confirmation of patient identity (*p* <0.0001) and hand hygiene improved significantly from baseline to follow-up (*p* = 0.0056; Table 2). While not statistically significant, observations of injection sites cleaned prior to therapeutic procedures saw improvement from baseline (48.7%) to follow-up (75.2%; *p* = 0.5154; Table 2; Fig 2).

**Table 2:** Combined pre- and post-training assessment of safe injection practice affecting patient safety.

	Pre		Post		p-value
	No.	%	No.	%	
**Diagnostic Observations**					
Patient identification confirmed	18	62.0	29	70.7	0.4849
Hand hygiene performed	5	17.2	26	63.4	0.0012
Injection site cleaned	27	93.1	17	100	0.2786
Sterile needle/syringe used	26	100	31	93.9	0.4842
**Therapeutic Observations**					
Patient identification confirmed	34	40.4	98	73.6	<0.0001
Hand hygiene performed	19	22.0	108	80.6	0.0056
Injection site cleaned	39	48.7	88	75.2	0.5154
Sterile needle/syringe used	85	98.8	128	97.7	0.6127

Abbreviation: No. = Number.

### Behaviors affecting HCW safety

The percentage of HCW who immediately disposed of a needle following a diagnostic procedure significantly decline from 96.5% to 92.5% (p = 0.0030; Table 3). We did not detect any other significant changes in safety practices at follow-up.

**Table 3:** Pre- and post-training assessment of safe injection practices affecting healthcare worker safety.

	Pre		Post		p-value
	No.	%	No.	%	
**Diagnostic Observations**					
Needle recapped after use	8	38.1	23	57.7	0.4918
Needle disposed immediately	28	96.5	37	92.5	0.0030
Sharps container located within arm’s reach	22	81.4	28	68.2	0.2724
**Therapeutic Observations**					
Needle recapped after use	57	68.7	79	61.7	0.5437
Single hand recapping technique	22	43.1	54	68.3	0.1312
Needle disposed immediately	63	78.7	109	84.5	0.1414
Sharps container located within arm’s reach	29	36.7	90	78.2	0.0635
Sterile needle/syringe used	85	98.8	128	97.7	0.6127

Abbreviation: No. = Number.

### Healthcare worker knowledge and attitudes

We did not detect any statistically significant changes in HCW knowledge of diseases transmitted via injections or supervisory practices between baseline and follow-up (Table 4). While not statistically significant, HCW knowledge of the transmission of HIV and HCV through unsafe injection practiced decreased from 100% to 92.9% and 61.5% to 57.7%, respectively. HCW knowledge of HBV transmission via unsafe injection practices increased from 79.4% to 90.1%, however, this increase was not seen as statistically significant. The availability of HIV post-exposure prophylaxis (PEP) for needle stick injuries declined from 60.0% to 53.1%.

**Table 4:** Pre- and post-training assessment of healthcare worker knowledge and attitudes.

	Pre		Post		
	No.	%	No.	%	p-value
*Injection provider*					
Knowledge of diseases transmitted	39	100	66	100	-
Human immunodeficiency virus	39	100	66	92.9	0.2742
Hepatitis B virus	31	79.4	64	90.1	0.1768
Hepatitis C virus	24	61.5	41	57.7	0.6333
Ever experienced needle-stick injury	11	28.2	24	34.2	0.5714
PEP* Available	21	60.0	34	53.1	0.7748
Received injection safety training	19	50.0	26	37.1	0.3300
*Injection provider supervisor*					
Reminder- Hand Hygiene	13	50.0	14	58.3	0.4355
Reminder- Do not recap needles	6	23.0	4	16.6	0.6503
Reminder- Careful of NSI*	6	23.0	8	33.3	0.4748
Needle Stick Injury	6	23.0	5	21.7	0.8892
PEP* Available	14	56.0	11	45.8	0.7164

**Abbreviation:** PEP = post exposure prophylaxis; NSI = Needle Stick Injury.

## Discussion

This paper presents data from a pre/post evaluation of an “Injection Safety Training” intervention implemented to improve injection safety practices among HCWs in Cambodia. We found that the injection safety training intervention had the capacity to improve hand hygiene practice, patient identification, and immediate needle disposal significantly.

Injection safety is a major issue in many low resource settings including countries in the region such as Nepal, India, China, and Pakistan [8,19–21]. The need for training and sensitization of best practices for HCWs for safe injection practices has been a priority for WHO’s “Safe Injection” campaigns for over a decade and has been highlighted in studies conducted in the region [21,22]. A study in India found that public sector facilities had more unsafe injection practices than that of private sector [20]. The major issues identified with HCW unsafe injection practices in multiple countries can be categorized as those factors that affect patient safety (needle reuse and hand hygiene), those that affect HCW safety (recapping needles, needle disposal), and factors that harm the community (waste management) [1,20].

### Factors affecting patient safety

Alcohol-based hand rub has been proven to reduce bacterial microflora of hands, increase hand-washing adherence and frequency, and decrease the occurrence of nosocomial infections [23–25]. Our findings were similar to other studies that have documented that only 50–70% of HCWs comply with hand-hygiene recommendations following training interventions. [26]. Multiple studies have elucidated the reasons for poor hand hygiene practices to lack of training, access to hand hygiene facilities and supervision to name a few [27,28].

Improvement was observed with injections sites cleaned prior to commencement of diagnostic procedures. Few prior studies have elaborated on the practice of cleaning of injection sites prior to administration of injections, highlighting the need to capture such information in future investigations[19,20,29,30].

### Factors affecting provider safety

The use of safety boxes for disposal of used syringes is important for the safety of HCWs and other staff, e.g., cleaners. In our study, we found that proper disposal of sharps significantly declined for diagnostic procedures between baseline and follow-up. This could have been due to interruptions in supplies or other management related issues. Prior studies in India and Nepal have shown a relationship between stocks outs and poor disposal of needles and syringes [6,18]. While disposal of sharps during therapeutic procedures improved slightly between baseline and follow-up, this increase was non-significant.

NSI are one of the biological hazards associated with injection use. The practice of needle recapping, and the technique used (single handed versus two handed) did not show significant improvement post-training in our study. A similar study in India saw 17% of HCW recapping needles after use which was far lower than our findings of 68.7% (baseline) and 61.7% (follow-up) [20].

In this study, the number of injection providers who reported having ever received a needle stick injury increased from baseline to the post-training follow-up, however, this was not statistically significant. This finding could be attributed to poor diffusion of knowledge through the training cascade, poor mentorship and reinforcement of best practices or lack of auto-disabled needles and syringes [10,20]. These findings are similar to a prior study conducted in Cambodia, where 53% of service provider NSIs in the last 12 months [20,30]. However, these findings were lower than that seen in similar studies conducted in the region such as in Nepal (56%) and Pakistan (33%) [8,30].

HCWs have a high risk of occupational exposure, more so in low-resource countries, with high incidence of blood borne diseases and unsafe practices. The risk of HCWs acquiring a blood borne pathogen after occupational exposure depends on multiple factors such as prevalence of infection in specific population; frequency of activities capable of transmitting the infectious agent; and the availability and efficacy of PEP [31]. Availability of PEP as reported by injection providers decreased from pre- to post-training follow-up. However, the lack of PEP availability was similar to that seen in another study in India where it was 60% [32].

HCW knowledge of diseases that are transmissible via unsafe injection safety practice is a crucial component of best practices. Injection provider awareness that some diseases are transmissible via NSI remained constant between baseline and follow-up. This is comparable to HCW knowledge in China that unsafe injection practice were associated with HIV and HBV transmission (95% and 89%, respectively), with 59% not recognizing that HCV was a potential risk [33]. However, there was variation in HCW knowledge at baseline and post-training assessments concerning HIV and HCV being transmissible via unsafe injection practice. It is possible that this change was due to public health awareness campaigns that might have occurred during the period prior to the follow-up survey. Additionally, given the complexity of the workforce and the delays in cascading training it was difficult to evaluate if the change in knowledge is a result of poor training versus lack of training.

The cascade model is often used in low resource settings for multiple reasons such as: cost effectiveness; rapid dissemination of knowledge; increased satisfaction; and improved skill retention [12,34]. Despite, the improvement seen in some aspects of injection safety, the Injection Safety Training intervention was subject to problems associated with a “cascade model” that have been well documented in prior studies such as incomplete diffusion of knowledge, the need for training to be reinforced with mentoring, and potential interruptions in supplies [18,35]. To help mitigate some of the known issues with the cascade model, the intervention was implemented using experts in the field of injection safety, who also understood the local culture, and conducted trainings in the local language [34]. The intervention was designed in collaboration with the Cambodian MOH to ensure that the existing structure of district supervisors could continue to provide re-enforcement and mentorship for best practices.

### Lessons learned and future challenges

The objective of this evaluation was to measure the impact of the “Injection Safety Training” intervention. The following lessons were learned: (1) a crucial element of the strategy to improve injection safety and practice involved ensuring all HCWs were trained. Unfortunately, the rollout of the cascade training did not result in all HWCs being trained at the time of the follow-up assessment. (2) Follow-up field visits after the training had not been accounted for in the training model. (3) The use of the right trainer and training in local language was a key factor in the intervention’s success. (4) This training model has since been integrated into existing in-service and pre-service training programs. (5) The intervention did not engage policy makers or those involved in the procurement of safe injection equipment, and this may have contributed to the reuse of injection equipment that were observed during the end line assessment. Previous studies have shown that sustainable solutions, require multidisciplinary management decisions and not just behavioral and knowledge interventions; instead to ensure long term impact system wide changes such as improved supply chain, and improved supervision and monitoring also need to be incorporated into these interventions [36].

### Limitations of the study

First, the findings from this evaluation are not representative of the entire country. The baseline and follow-up assessments were conducted in a sample of health facilities in two provinces. Second, the study did not match pre assessment participants to that of post-test participants. Third, the injection safety-training cascade was rolled out to some provinces; and, at the time of the post-training follow-up, not all HCWs across the country or at the surveyed health facilities had received the training. Fourth, respondents may respond/behave differently if they are watched or a study is being done on their activity. This changed response is called the Hawthorne effect [37]. Fifth, the real-time translation of the questionnaire from English to Khmer may have resulted in questions being translated both inconsistently and inaccurately. Lastly, factors such as availability of sterile supplies and reassignment of trained HCWs were not taken into account during the analysis.

## Conclusion

We found significant improvements in the practice of hand hygiene and immediate disposal of sharps in the post-training follow-up but not in the awareness of HIV and HCV as blood borne pathogens transmissible via unsafe injection practice. However, these findings are not representative of all HCWs in the country. Further pre-service and in-service training and monitoring of injection safety practices are necessary to sustain and build upon the behavior change brought about by the training intervention.
